# Dr. Lewis Barnes

**Published:** 1908-08

**Authors:** 


Dr. Lewis Barnes died of apoplexy at his home in Oxford, Conn., July 5, 1908, aged 84 years. He graduated from the medical department, University of Buffalo, in 1851.

